# Combined Acupoint Massage and Abdominal Mirabilite Application for Accelerating Gastrointestinal Recovery in Pediatric Patients After Endoscopic Retrograde Cholangiopancreatography: Protocol for a Randomized Controlled Trial

**DOI:** 10.2196/87961

**Published:** 2026-02-03

**Authors:** Xiaowei Pu, Shan Wang, Chen Cui, Libing Liang, Qunsong Shen, Wei Luan, Caiqin Wu

**Affiliations:** 1 Department of Pediatrics Shanghai University of Traditional Chinese Medicine Affiliated Shuguang Hospital Shanghai, Shanghai China; 2 School of Nursing Shanghai University of Traditional Chinese Medicine Shanghai, Shanghai China; 3 Department of Nursing Shanghai University of Traditional Chinese Medicine Affiliated Shuguang Hospital Shanghai, Shanghai China

**Keywords:** acupoint massage, mirabilite, pediatric, endoscopic retrograde cholangiopancreatography, ERCP, gastrointestinal function, postoperative recovery, randomized controlled trial

## Abstract

**Background:**

Endoscopic retrograde cholangiopancreatography (ERCP) is crucial for managing pediatric hepatobiliary diseases but frequently results in postoperative gastrointestinal dysfunction such as delayed flatus and defecation, which can prolong recovery and increase the risk of complications. Nonpharmacological interventions such as acupoint massage and mirabilite application offer potential benefits, but evidence for their efficacy and synergy in children after ERCP is lacking.

**Objective:**

This study aims to evaluate the individual and combined effects of acupoint massage and abdominal mirabilite application on accelerating gastrointestinal recovery in pediatric patients following ERCP.

**Methods:**

A single-center, single-blind, randomized controlled trial will be conducted involving 72 children aged between 2 and 12 years who have undergone ERCP. Participants will be randomly allocated to 1 of the 4 groups: acupoint massage group (stimulating ST-36, ST-25, CV-12, and PC-6 for 2 minutes per point; twice daily), mirabilite group (abdominal application for 1 hour; twice daily), combination group (both interventions; at least 1 hour apart), or conventional care group. The primary outcome is the time to first postoperative flatus and defecation. The secondary outcomes include the frequency of postoperative nausea and vomiting and inflammatory markers. Data will be analyzed using intention-to-treat analysis with SPSS, using ANOVA, chi-square, and nonparametric tests as appropriate.

**Results:**

This trial is currently in progress, having received funding in January 2023 and July 2023. Participant recruitment commenced on November 1, 2024, with an anticipated completion date of October 30, 2026. As of January 2026, a total of 38 pediatric patients have been enrolled and randomized, while data collection and intervention delivery are ongoing. No interim analysis has been conducted. The final analysis will begin after recruitment and follow-up are complete, with primary results expected to be published in the second half of 2026.

**Conclusions:**

This study is the first to rigorously assess the efficacy and potential synergistic effects of acupoint massage and mirabilite application for enhancing gastrointestinal recovery after pediatric ERCP. Positive findings could establish a safe, nonpharmacological protocol to improve postoperative outcomes, reduce hospital stays, and minimize complications in this vulnerable population.

**Trial Registration:**

International Traditional Medicine Clinical Trial Registry ITMCTR2025000670; https://itmctr.ccebtcm.org.cn/mgt/project/view/3385640124071694411

**International Registered Report Identifier (IRRID):**

DERR1-10.2196/87961

## Introduction

### Background

Endoscopic retrograde cholangiopancreatography (ERCP) is a significant minimally invasive diagnostic and therapeutic procedure extensively used in the diagnosis and treatment of pediatric hepatobiliary disorders, including common bile duct stones, biliary strictures, biliary fistulas, and chronic pancreatitis [1-3]. Research indicates that children undergoing ERCP experience varying degrees of postoperative gastrointestinal dysfunction, including delayed passage of flatus and stool, abdominal distension, nausea, vomiting, and diminished bowel sounds [2,4]. If not promptly addressed, these complications may lead to severe outcomes, such as intestinal perforation and pancreatitis [5]. Postoperative gastrointestinal dysfunction not only increases the child’s discomfort but may also prolong hospital stays, raise intravenous nutrition requirements, and heighten the risk of complications, ultimately impacting the overall recovery process and exacerbating the economic burden on health care systems [6,7].

The mechanisms contributing to gastrointestinal dysfunction following ERCP are multifactorial, encompassing preoperative anxiety, opioid use, mechanical stimulation of the intestines from surgical procedures, vagal nerve dysfunction, and postoperative inflammatory responses [8-10]. Furthermore, the gastrointestinal development of children is incomplete, and their metabolic and neuroregulatory functions are more sensitive than those of adults, potentially exacerbating the delay in postoperative gastrointestinal recovery [11,12]. Current postoperative management strategies have shown limited effectiveness in the pediatric population. For instance, the administration of low-dose opioids after gastrointestinal surgery fails to alleviate postoperative pain and is associated with an increased incidence of postoperative gastrointestinal dysfunction [9]. Currently, there is no efficient, safe, and adherence-friendly specific protocol specifically designed to enhance gastrointestinal function in children after ERCP, highlighting the pressing need to explore nonpharmacological, low-risk alternative therapies.

Traditional Chinese medicine (TCM) posits that postoperative gastrointestinal dysfunction is categorized as “abdominal distension” or “constipation,” with the underlying pathogenesis frequently associated with Qi stagnation and bowel obstruction [13-15]. Acupoint massage stimulates specific acupoints to unblock meridians and harmonize Qi and blood, thereby enhancing gastrointestinal motility [16,17]. Research has demonstrated that massage activates parasympathetic nerve activity, promoting intestinal movement [18]. The topical application of mirabilite (sodium sulfate) aligns with TCM’s principle that “external treatment corresponds with internal treatment.” This substance possesses a cold nature and salty flavor, allowing it to clear heat, reduce swelling, and soften hardness to disperse masses; its high osmotic properties alleviate edema of the intestinal wall and stimulate local nerve reflexes, thus accelerating the recovery of bowel function [19]. The integration of these 2 methods creates a “comprehensive internal and external treatment” model; massage regulates the overall organ function and Qi, while mirabilite addresses localized swelling and constipation. This approach is advantageous due to its noninvasive nature, the ease of application, and cost-effectiveness, making it particularly suitable for pediatric patients who are intolerant to medication.

Research on adults has confirmed the effectiveness of the topical application of mirabilite in enhancing gastrointestinal function and reducing inflammation following laparoscopic cholecystectomy and laparoscopic appendectomy for mild acute biliary pancreatitis [20,21]. Additionally, acupoint massage has been shown to improve gastrointestinal function in patients undergoing gynecologic laparoscopic procedures and gastrointestinal surgery [17,18]. Despite the potential demonstrated by single therapies in adult studies, there is a significant lack of high-quality evidence supporting the use of acupoint massage in conjunction with the topical application of mirabilite to enhance gastrointestinal function in children following ERCP. Existing research not only lacks evidence for the effectiveness of acupoint massage, topical application of mirabilite, and their combined interventions for improving gastrointestinal function following ERCP in children but also fails to standardize intervention parameters related to gastrointestinal recovery in this population, such as the choice of acupoint for massage, duration of the procedure, and dosage of mirabilite. We hypothesize that all 3 intervention strategies will shorten the recovery time of gastrointestinal function and reduce postoperative adverse reactions compared to standard care, and the combined group is expected to yield the best results.

This protocol uses a 4-group parallel control design, comprising an acupoint massage group, a mirabilite external application group, a combined intervention group, and a routine care group. The primary objective is to compare the independent and synergistic effects of acupoint massage and mirabilite external application on the recovery of gastrointestinal function, specifically the time to first flatulence and defecation following pediatric ERCP. The secondary objective is to clarify the differences in the effects of various interventions on secondary outcomes, including the frequency of postoperative nausea and vomiting (PONV), as well as inflammatory markers, such as serum amylase and white blood cell (WBC) count. Additionally, the operational standards, including massage techniques, the duration of mirabilite application, and the evaluation processes of interventions, will be standardized to ensure methodological rigor, thereby providing a reliable basis for subsequent clinical translation.

### Study Aims

This study aims to comprehensively evaluate the effects of acupoint massage, the external application of mirabilite, and their combined intervention on gastrointestinal function recovery in children following ERCP procedures. The primary objectives include comparing the efficacy of the 3 interventions—acupoint massage, mirabilite application, and the combined approach—against a routine care group, specifically in terms of shortening the time to first postoperative flatus and bowel movement. Additionally, this study aims to clarify whether the combined intervention offers advantages over individual therapies to determine the optimal clinical strategy. The secondary objectives involve assessing the degree of improvement in postoperative symptoms, including PONV; analyzing changes in inflammatory markers, including serum amylase and WBC count; and verifying the safety of the interventions regarding issues such as skin irritation and procedural tolerance. This research not only determine the optimal strategy to address the existing evidence gap in accelerated recovery following pediatric ERCP but also provides a scalable, standardized protocol for rapid postoperative recovery in pediatric patients.

## Methods

### Study Design

**Figure 1:**
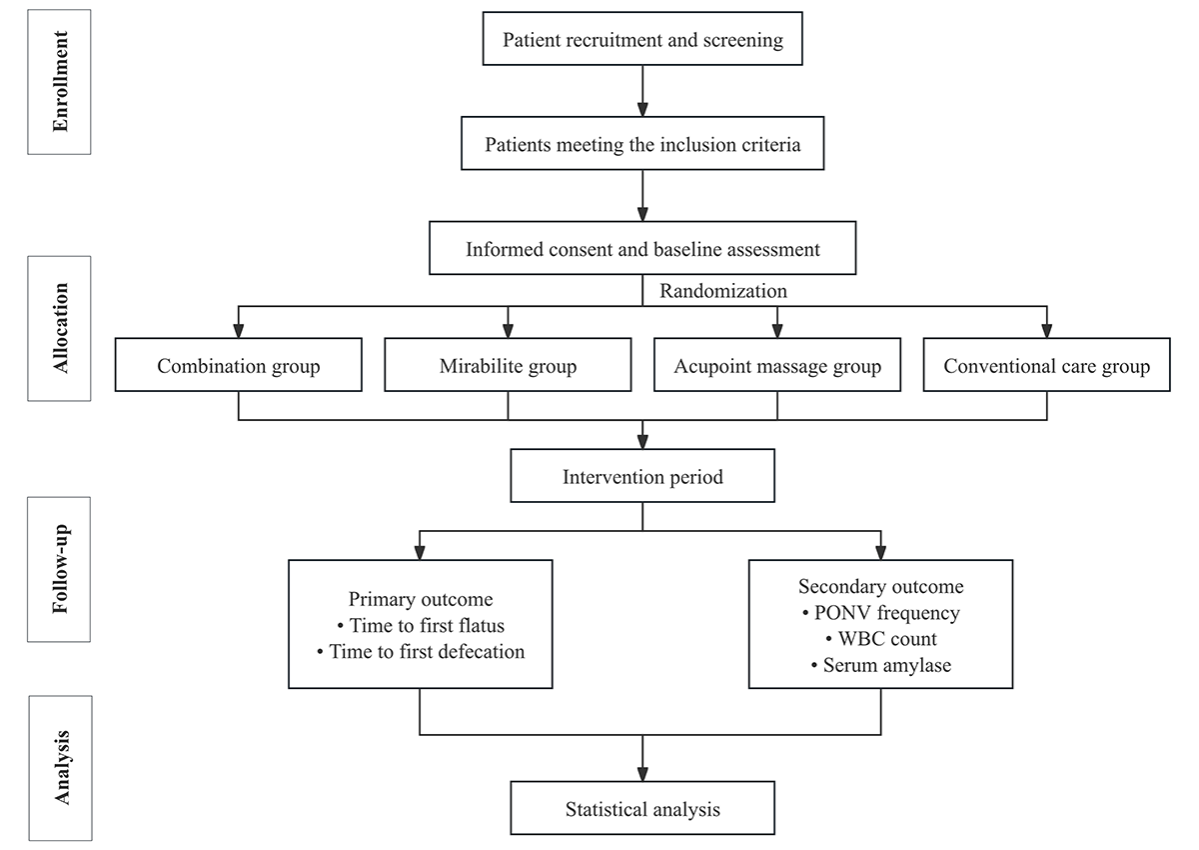
Flow diagram of the study design. PONV: postoperative nausea and vomiting; WBC: white blood cell.

A parallel 4-group, single-blind, randomized controlled trial (RCT) will be conducted from November 1, 2024, to October 30, 2026. The study will take place in the pediatric department of Shuguang Hospital in Shanghai. After baseline assessments, eligible participants will be randomly assigned in a 1:1:1:1 ratio to 4 groups: acupoint massage group, mirabilite group, combination group, and conventional care group (Figure 1; Table 1). Table 2 presents the content and procedures used in each intervention group. The standardized intervention manual can be found in Multimedia Appendix 1. The primary outcome measure will be the time to postoperative gas passage or stool, whereas the secondary outcomes will include the incidence of PONV as well as changes in inflammatory markers. This study will adhere to the SPIRIT (Standard Protocol Items: Recommendations for Interventional Trials) guidelines (Multimedia Appendix 2) [22]. This protocol was registered at the International Traditional Medicine Clinical Trial Registry (ITMCTR2025000670) on April 7, 2025.

**Table 1:** Clinical trial procedure.

Study period						Enrollment			Baseline (before surgery)			Intervention and assessment (after surgery)			Closeout (discharge)
**Enrollment**															
	Eligibility screen					✓									
	Informed consent					✓									
	Randomization											✓			
**Interventions**															
	Combination intervention											✓			
	Mirabilite application											✓			
	Acupoint massage											✓			
	Routine care								✓			✓			
**Assessments**															
	**General social demographic data**					✓									
		**Primary outcomes**													
			Time to first flatus							✓			✓		
			Time to first defecation							✓			✓		
		**Secondary outcomes**													
			Postoperative nausea and vomiting frequency							✓			✓		
			White blood cell count				✓			✓			✓		
			Serum amylase				✓			✓			✓		
	**Safety monitoring**														
		Skin irritation									✓			✓	
		Massage tolerance									✓			✓	

**Table 2:** Intervention program.

Group	Intervention components	Frequency or duration	Key operational parameters	Safety monitoring
Acupoint massage	Stimulation of Zhongwan (CV-12), Tianshu (bilateral; ST-25), Zusanli (bilateral; ST-36), and Neiguan (bilateral; PC-6)	Twice daily (2 minutes per acupoint)	Technique: rotatory kneading+vertical pressing Pressure: ≤0.5 kg (aged 2-5 y) and ≤1 kg (aged 6-12 y) Sequence: CV-12→ST-25→ST-36→PC-6	Preprocedure wound assessment Real-time tolerance monitoring Skin response check Emergency stop for vomiting or pain
Mirabilite application	Periumbilical paste application (xiphoid process to pubic symphysis)	Twice daily (1 hour, with a 5-minute margin on either side)	Allergy test Preparation: 1:1 mirabilite: water (36 °C-38 °C) Thickness: 0.5 cm uniform layer Fixation: hypoallergenic tape Constant temperature heating pack	Preapplication skin test Temperature monitoring (36 °C-38 °C; ΔT≤1 °C) Visual inspection Discontinuation for erythema or blisters
Combination group	Both interventions	Massage: 2 per day; mirabilite: 2 per day; minimum interval: 60 minutes	Sequence: massage→mirabilite (preferred) Prohibited: massage within 1 hour after mirabilite application Separate documentation for both	Combined monitoring of both interventions

### Participants

Participants will be recruited from children who have undergone ERCP in the pediatric department of Shanghai Shuguang Hospital. Inclusion criteria are as follows: (1) aged between 2 and 12 years, (2) having completed ERCP, (3) deemed suitable for pediatric acupressure and external mirabilite application, (4) having provided written informed consent by legal guardians, and (5) having provided age-appropriate assent from the child. Exclusion criteria are as follows: (1) history of abdominal surgery or severe gastrointestinal dysmotility; (2) concomitant failure of vital organs, such as the heart, lung, or kidney; (3) known allergy to mirabilite or skin lesions contraindicating topical application; (4) cognitive or behavioral impairment that would compromise cooperation with acupressure; and (5) intraoperative conversion to open surgery due to major complications, such as perforation or severe bleeding. Participants will be withdrawn under the following circumstances: (1) voluntary withdrawal by the child or their parent; (2) loss of communication during the intervention that precludes further assessment; or (3) the occurrence of a serious adverse event, including marked clinical deterioration or an acute medical condition.

Eligible children will be identified through an electronic medical record system, with 2 senior physicians independently evaluating their enrollment criteria. In the event of disagreement, a third expert will adjudicate. All participants will have the research protocol thoroughly explained by a dedicated research nurse before surgery, and informed consent will be obtained from the guardians and the children.

### Sample Size

G*Power (version 3.1; Heinrich Heine University Düsseldorf) software was used to calculate the sample size. We used the *F* test as the method for statistical analysis, establishing an α of .05 and a power (1–β) of .95. The estimation was based on data from a previous high-quality clinical trial, which used a 3-arm parallel design evaluating combined mirabilite application with massage, massage alone, and mirabilite application alone in patients undergoing laparoscopic hepatectomy [23]. Using the reported group means, a pooled SD of 13.03 hours, and a sample size of 30 per group from that trial, the software estimated an effect size (*f*) of 0.593. On the basis of this effect size, the calculated total sample size required for this 4-group study was 56 participants. To account for an anticipated dropout rate of 20%, the final planned total sample size is 72 participants, with approximately 18 (25%) individuals allocated to each group.

### Randomization and Blinding

This study uses a randomized block design with a block length of 4. Random numbers for grouping were generated using Stata (version 18.0; StataCorp). A dedicated researcher, who will not participate in the recruitment of participants or data collection, will oversee the random grouping process, thereby ensuring that the grouping decisions remain independent of actual operations. Before the initiation of the study, personnel not involved in participant recruitment or data collection will create an allocation table based on the random number sequence and maintain this table in strict confidentiality to prevent any potential leaks. Group allocations will be concealed within sealed, opaque envelopes, which the researcher will open before the intervention to access the allocation plan for each patient.

Given the overt nature of acupoint massage and external mirabilite application, blinding of participants, their guardians, and the health care providers administering the interventions was not feasible. This represents an inherent methodological limitation of this study. To mitigate potential measurement bias due to the lack of blinding, we implemented several procedural strategies. A strict and standardized outcome assessment protocol was established, wherein independent assessors, specifically nurses who were blinded to group allocation, evaluated patients at 4-hour intervals. These evaluations were conducted through direct auscultation of bowel sounds combined with patient or parental reporting. Documentation of first flatus or defecation requires both active bowel sounds and clear verbal confirmation, thereby reducing sole reliance on subjective reporting. Researchers responsible for data collection and statistical analysis remained blinded to group assignment and accessed only deidentified datasets to maintain analytic independence. Furthermore, throughout the informed consent process and study duration, families received standardized, neutral communication, emphasizing that the study aimed to compare different postoperative care approaches, avoiding implications of differential efficacy to reduce expectation bias. All outcomes were recorded on structured case report forms (CRFs), mandating concurrent documentation of objective signs, such as auscultation findings, alongside the reporting source.

### Interventions

#### Conventional Care Group

The children will receive standard postoperative care, including monitoring of vital signs, necessary medication, as-needed pain relief, dietary adjustments, and rehabilitation training, but will not undergo acupoint massage or the external application of mirabilite nor will any investigational interventions be administered.

#### Acupoint Massage Group

On the basis of conventional care, standardized acupoint massage will be performed by certified physicians and nurses to promote the recovery of organ function and alleviate symptoms of gastrointestinal dysfunction. The selected acupoints include Zusanli (bilateral; ST-36), Tianshu (bilateral; ST-25), Zhongwan (CV-12), and Neiguan (PC-6), which are informed by pertinent studies on the recovery of gastrointestinal function following laparoscopic surgery and adhere strictly to the “World Health Organization Standard Acupuncture Point Localization” [24,25]. Each acupuncture point will receive a combination of kneading and pressure techniques at a frequency of 30 repetitions per minute, with a pressure depth ranging from 0.5 to 1 cm, sustained for a duration of 2 minutes, and administered twice daily. The applied pressure must remain within a tolerable range for the child, ensuring that the skin becomes slightly reddened without eliciting pain. The child’s abdominal wound will be assessed before the procedure to avoid the puncture site.

#### Mirabilite Group

On the basis of conventional care, an external application of mirabilite will be administered to the abdomen, drawing on relevant studies concerning acute pancreatitis [26]. Preparation entails grinding medical-grade mirabilite into a powder, which is then mixed with warm water in a 1:1 ratio to create a paste. This paste will be evenly spread onto gauze to a thickness of approximately 0.5 cm and placed over the area surrounding the navel, extending from above the xiphoid process to below the pubic symphysis. The gauze will be anchored with breathable tape to prevent pressure on the drainage tube, while a constant temperature heating pack will maintain warmth during the application. Each application will last for 1 hour, administered twice daily. Skin reactions will be closely monitored, and the application will be discontinued immediately if redness or allergic reactions occur.

#### Combination Group

This group will simultaneously receive both acupoint massage and external application of mirabilite, adhering to the same protocols as the single-intervention groups, but spaced apart from each other. An interval of at least 1 hour will be maintained between the 2 interventions to avoid interference. The acupoint massage will promote the smooth flow of Qi and blood within the meridians, while the localized stimulation from the external application of mirabilite will further facilitate the recovery of gastrointestinal function and enhance therapeutic effects.

### Outcome Measures

#### General Sociodemographic Data

To characterize the study population and assess potential confounding factors, we will collect basic demographic and clinical information from pediatric patients before the intervention, including age, gender, height, weight, and BMI, as well as disease diagnosis, specific indications for undergoing ERCP, history of abdominal surgeries, comorbidities, relevant family medical history, the number of previous ERCP procedures, guardians’ educational level, annual family income and the type of residence (urban or rural). Annual family income will be categorized as: <CNY ¥50,000 (US $7183), CNY ¥50,000-100,000 (US $7183-14,366), CNY ¥100,000-200,000 (US $14,366-28,731), and >CNY ¥200,000 (US $28,731).

#### Primary Outcomes

The primary objective of this study is to investigate the speed of gastrointestinal function recovery in pediatric patients following ERCP. The core indicators include recording and comparing the time until the first passage of gas and the time until the first bowel movement after surgery between the 2 patient groups. Assessments will be performed every 4 hours by trained nursing staff through direct inquiry of the child or guardian, combined with auscultation of bowel sounds, to objectively validate the occurrence of flatus or defecation. These 2 time points serve as critical objective markers that reflect the recovery of gastrointestinal motility.

#### Secondary Outcomes

##### Overview

To comprehensively evaluate postoperative recovery and investigate potential mechanisms, the subsequently mentioned secondary outcomes will be monitored. The first outcome is the incidence of PONV. The frequency of PONV episodes will be recorded every 4 hours. The second outcome is postoperative inflammatory markers. To establish baseline values and track the perioperative inflammatory trajectory, venous blood samples for quantification of WBC count and serum amylase levels will be collected before surgery, at 3 hours after surgery, and on the morning of postoperative day 1. These parameters will serve to assess pancreatic functional recovery, screen for complications, and gauge the magnitude of the systemic inflammatory response. Furthermore, they will provide indirect indicators of gastrointestinal functional status, offering valuable reference data for monitoring postoperative clinical progression.

##### Adherence

To ensure strict adherence to the intervention plan, researchers will actively communicate with both the parents and the child to confirm the timing of the intervention, thereby ensuring that participants can attend on time. Additionally, the child will receive a gift upon enrollment and again at the conclusion of the final follow-up to enhance motivation and engagement throughout the study.

##### Data Collection and Management

Data collection will be conducted by trained, blinded researchers. Baseline data will be collected after the children have completed random allocation and before the initiation of the first intervention. Data will be meticulously recorded during each acupoint massage session and the application of mirabilite, which includes specific time points of the intervention, actual duration of execution, selected acupoints for acupressure, intensity of techniques, site of mirabilite application, and the child’s tolerance and compliance with the intervention. Core observational indicators for postoperative recovery will be collected strictly, with the times of first flatus and first bowel movement recorded through scheduled inquiries by medical staff to the child or family, validated through physical examinations, and accurately documented. Instances of PONV will also be recorded, while inflammatory indicators will be documented at 3 hours after surgery and the following morning.

All collected data will be recorded using standardized paper CRFs. All paper CRFs will be securely stored by the principal investigator, and data entry will follow a double-entry system. An electronic database will be established using Microsoft Excel, with regular checks to identify potential errors or outliers. Source data verification will be performed regularly, comparing CRF data with original medical records to ensure transcription accuracy.

##### Quality Control and Precautions

The research team developed a comprehensive standard operating procedure in advance, clearly delineating the intervention specifics related to acupoint massage and the external application of mirabilite. Before the formal commencement of the study, all intervention researchers must undergo unified theoretical and practical training, focusing on controlling the intensity of the technique and accurately locating acupoints. Consistency assessments will ensure a high degree of homogeneity and reproducibility in intervention measures among different practitioners.

Safety monitoring will be conducted continuously throughout the intervention process. During each acupoint massage session, the operator must assess and document the child’s tolerance to the treatment in real time, with particular attention to controlling the acupressure intensity to minimize discomfort or injury. Before, during, and after each application of mirabilite, skin reactions in the treated area must be closely monitored and documented, emphasizing any allergic or irritative symptoms, such as rashes, itching, blisters, or ulceration, as well as the child’s reported discomfort. Any adverse events or suspected adverse reactions, irrespective of their relation to the research intervention, must be recorded promptly and thoroughly according to the protocol, detailing the time of occurrence, manifestations, severity, duration, management measures, and outcomes.

Emergency response plans have been pre-established to address potential safety issues. To manage possible skin allergic reactions resulting from the external application of mirabilite, emergency medications will be readily available on-site for localized treatment; should a clear allergic reaction or severe skin irritation arise, the application of mirabilite will be immediately terminated, symptomatic treatment will be administered, and adverse events will be reported as required. If a child shows discomfort, distress, or verbal or behavioral refusal during an intervention, the procedure will be paused immediately. The researcher will comfort the child and consult with the guardian. The intervention may resume only if the distress is transient and the child and guardian agree. Continued refusal or significant distress will lead to termination of that session or withdrawal from the study. All research personnel must be thoroughly familiar with and strictly adhere to these safety protocols and emergency plans, prioritizing the safety of the children involved and promptly reporting any incidents that exceed the prescribed protocols or severe adverse events to the principal investigator and the ethics committee.

### Data Analysis

Statistical analyses will be performed using SPSS (version 25.0; IBM Corp) and RStudio (Posit PBC), incorporating the *survival* package. A 2-sided significance level of .05 will be applied for all tests, and an intention-to-treat analysis will be implemented to handle missing data.

Categorical baseline variables will be summarized as frequencies and percentages, with group comparisons conducted using chi-square tests or Fisher exact tests as appropriate. Continuous variables will be described as mean with SD if normally distributed and compared via 1-way ANOVA; nonnormally distributed variables will be presented as median with IQR and compared using the Kruskal-Wallis *H* test. Time-to-event analysis will be applied to the primary outcomes, namely time to first flatus and time to first defecation. Cumulative event rates will be visualized using Kaplan-Meier curves, and overall, between-group differences will be assessed via the log-rank test. To address multiple comparisons, a prespecified hierarchical contrast approach will be used. Primary contrasts, including each intervention group compared to the routine care group, will be evaluated using a Bonferroni-adjusted significance threshold of .0167. Exploratory pairwise contrasts between the combination group and each monotherapy group will be conducted only if significant differences are observed in the primary contrasts. Additionally, a Cox proportional hazards regression model will be used to estimate adjusted hazard ratios with 95% CIs for each intervention relative to routine care while controlling for baseline covariates identified in univariable analysis with a *P* value of <.10.

The secondary outcome analysis will include comparisons of PONV incidence using chi-square tests, with Bonferroni-adjusted pairwise testing where applicable. Inflammatory markers, including WBC count and serum amylase, will be analyzed using repeated-measures ANOVA to evaluate changes at 3 hours after surgery and on the morning of postoperative day 1. Safety outcomes will be summarized descriptively, and between-group differences in adverse events will be examined using chi-square or Fisher exact tests.

### Ethical Considerations

This study adheres to the Declaration of Helsinki and Chinese pediatric research ethics guidelines [27]. A structured, age-appropriate informed consent process will be implemented. Trained research nurses will explain the study purpose, procedures, risks, benefits, privacy measures, and the right to withdraw to the legal guardian. Written consent will be obtained after ensuring full understanding.

We place the highest priority on respecting the child’s own will. In accordance with established pediatric ethical guidelines, a tiered assent process will be applied [27]. Children aged between 8 and 12 years will receive a simplified explanation using age-appropriate language and visual aids, and those who demonstrate understanding will be invited to sign a child assent form. For children aged between 6 and 8 years, verbal assent will be sought using simple language, and their agreement will be formally documented. For children aged between 2 and 6 years, participation will be based primarily on guardian consent; however, researchers will continuously monitor the child’s emotional and behavioral responses. Persistent distress or clear refusal will be respected as an expression of dissent, leading to immediate pausing or adjustment of the procedures. The child’s right to refuse is absolute. Regardless of guardian consent, if a child explicitly refuses or withdraws assent at any point, their decision will be honored without exception, resulting in nonparticipation or withdrawal from the study.

Aas a token of appreciation for participation and to support ongoing engagement, each child will receive a small, age-appropriate gift upon enrollment and upon completion of the final follow-up assessment. This compensation is not contingent on study outcomes and is provided to acknowledge the time and contribution of the participants and their families.

To protect participant privacy, all data will be anonymized using a unique study ID number. The linkage file matching study IDs to personal identifiers will be encrypted and stored separately, with access restricted to authorized study personnel only. Sensitive information, such as socioeconomic data, will be collected solely for aggregated analysis and will be reported in grouped categories without any identifiable details. All study documents, including original paper records, consent forms, and the electronic database, will be stored securely both during and after the completion of the study. Paper materials will be kept in locked cabinets within designated hospital areas, and electronic files will be maintained on password-protected, access-controlled hospital servers. Data retention will comply with all applicable ethical guidelines and regulatory requirements.

The protocol has been reviewed and approved by the ethics committee of Shanghai Shuguang Hospital (2024-1622-205).

## Results

This trial is currently in progress. This study received funding in January 2023 and July 2023. Participant enrollment commenced on November 11, 2024, with an anticipated completion date of October 30, 2026. Data collection and intervention delivery are actively ongoing. As of January 2026, a total of 38 pediatric patients have been successfully enrolled and randomized. No interim data analysis has been performed. The final data analysis is scheduled to commence upon completion of recruitment and follow-up, with primary study results expected to be available for publication in the second half of 2026.

## Discussion

ERCP is a minimally invasive procedure that is essential for the diagnosis and treatment of pediatric liver, biliary, and pancreatic diseases [28-30]. However, due to the immature gastrointestinal systems in children, gastrointestinal dysfunction following ERCP is common among pediatric patients, which increases the risk of complications and negatively impacts overall recovery [5,31,32]. Therefore, this study aims to evaluate both the independent and synergistic effects of acupoint massage combined with topical mirabilite on gastrointestinal recovery after ERCP in children using an RCT design. This research addresses the evidence gap in the field of rehabilitation following ERCP in children and provides new intervention options for pediatric enhanced recovery in surgical practice.

Both acupoint massage and the external application of mirabilite are grounded in a solid theoretical framework of TCM and are supported by preliminary evidence from modern medical research. TCM posits that postoperative gastrointestinal dysfunction falls within the categories of “distention” and “constipation,” attributing the core pathology to the obstruction of Qi and impaired digestive flow [33]. By stimulating Zusanli, Tianshu, Zhongwan, and Neiguan, the massage aims to unblock the meridians and harmonize Qi and blood, thereby restoring the function of visceral Qi and alleviating abdominal distention. Research involving patients following gynecologic laparoscopic surgery indicates that acupoint massage can significantly reduce the time to first bowel movement and the time to first gas passage while also regulating gastrointestinal hormone secretion, thereby facilitating the recovery of gastrointestinal function after surgery [16,34]. The external application of mirabilite adheres to the TCM principle that external treatments correspond to internal effects. It is cold in nature and salty in taste, capable of clearing heat, reducing swelling, softening hardness, and dispersing masses. Studies reveal that this application can shorten the recovery time of gastrointestinal function, alleviate postoperative gastrointestinal symptoms, reduce the hospital stay duration, decrease hospitalization costs, and does not increase the incidence of adverse events [21,23]. The efficacy of mirabilite in patients with sepsis combined with gastrointestinal dysfunction is evident, promoting gastrointestinal recovery, accelerating clinical improvement, and enhancing prognosis [35]. Relevant research suggests that mirabilite can exhibit anti-inflammatory and antiedematous effects and enhances gastrointestinal motility by stimulating the release of vasoactive intestinal peptide, substance P, and ghrelin [36]. In summary, acupoint massage and the external application of mirabilite have been implemented in adult postoperative care and have demonstrated efficacy, showing feasibility in both theoretical and clinical applications.

The core innovation of this study is the introduction of a combined intervention model that uses acupoint massage to regulate organ Qi and enhance gastrointestinal motility, alongside the external application of mirabilite to alleviate swelling and promote bowel movements. This integrated approach aims to create a synergistic effect that comprehensively supports the recovery of gastrointestinal function.

This research uses a rigorous RCT design, systematically evaluating the effects of acupoint massage, external application of mirabilite, and their combined approach on the improvement of gastrointestinal function in children after ERCP, which clearly distinguishes the independent contributions of each intervention and their potential synergistic effects, thus providing a reliable basis for determining the optimal treatment strategy. Furthermore, this study aims to establish standardized operational parameters for acupoint massage and the external application of mirabilite for children, including acupoint selection, techniques, treatment frequency, and duration for external application, laying the groundwork for future clinical applications. The research pays attention to clinical end point and biological indicators, aiming to provide preliminary evidence on the mechanisms of action of the interventions, especially concerning their anti-inflammatory effects. Additionally, rigorously recorded safety data that meet expectations will become crucial for supporting clinical promotion. This study has significant advantages, as a noninvasive, nonpharmacological external treatment method; its high safety and ease of operation make it more acceptable to children and parents. Compared to mandatory early ambulation, this method is less painful, with low costs for mirabilite and controllable labor costs, providing good health economic value.

This study has certain limitations that need to be considered. First, the research was conducted in a single center, which may lead to participant characteristics being confined to the patient population, surgical practices, and nursing routines of that center. Second, the highly observable nature of acupoint massage and mirabilite application made full blinding of participants, nursing staff, and families unachievable. While assessor blinding and a standardized outcome evaluation protocol incorporating objective signs, such as bowel sound auscultation, were used to reduce measurement bias, the awareness of group allocation among nurses could lead to implementation bias, and symptom reporting by families may be influenced by expectation bias. These factors may impact the reliability of key reported outcomes, specifically the timing of first flatus and defecation as well as the incidence of PONV. Additionally, while monitoring inflammatory markers and amylase levels provides some clues, the lack of direct measurement of vagal nerve activity or motilin levels limits a deeper understanding of the synergistic mechanism of the combined intervention.

Future research should concentrate on several crucial directions. First, it is essential to validate the reliability and generalizability of the results through multicenter, large-sample trials, thereby providing stronger evidence for the establishment of postoperative gastrointestinal recovery guidelines for pediatric ERCP. Second, researchers should deepen their exploration of underlying mechanisms by using gastrointestinal hormone monitoring and metagenomic analysis. Finally, it is important to broaden the applications of this strategy by investigating its effectiveness in other pediatric abdominal surgeries, ultimately benefiting a wider population of children.

In summary, this study aims to clarify the independent and synergistic effects of acupoint massage and mirabilite application on shortening gastrointestinal recovery time, alleviating related symptoms, and improving recovery processes in children after ERCP surgery through rigorous and standardized research. It aims to provide a safe, nondrug, child-friendly, and cost-effective option in clinical practice. This approach can not only optimize postoperative comfort for children, reducing hospitalization time and lowering the risk of complications and medical costs, but also provide critical evidence for developing standardized accelerated recovery pathways for pediatric ERCP. Furthermore, it promotes the scientific integration and application of TCM external treatment methods in modern pediatric perioperative care.

## Data Availability

The datasets generated or analyzed during this study are available from the corresponding author on reasonable request.

## Appendix

Multimedia Appendix 1 Postoperative application of mirabilite and acupoint massage after pediatric endoscopic retrograde cholangiopancreatography.

Multimedia Appendix 2 SPIRIT (Standard Protocol Items: Recommendations for Interventional Trials) checklist.

Multimedia Appendix 3 Peer review report by the Foundation of Shanghai University of Traditional Chinese Medicine (2023HLXL09, 23HLZX06).

## Abbreviations

CRF: case report form
ERCP: endoscopic retrograde cholangiopancreatography
PONV: postoperative nausea and vomiting
RCT: randomized controlled trial
SPIRIT: Standard Protocol Items: Recommendations for Interventional Trials
TCM: traditional Chinese medicine
WBC: white blood cell

## References

[1] AbiMansour JP, Martin JA (2024). Biliary endoscopic retrograde cholangiopancreatography. Gastroenterol Clin North Am.

[2] Baiu I, Visser B (2018). Endoscopic retrograde cholangiopancreatography. JAMA.

[3] Hosseini A, Sohouli MH, Sharifi E, Sayyari A, Sridharan K, Tajalli S, Imanzadeh N, Fatahi S (2023). Indications, success, and adverse event rates of pediatric endoscopic retrograde cholangiopancreatography (ERCP): a systematic review and meta-analysis. BMC Pediatr.

[4] Papaefthymiou A, Landi R, Arvanitakis M, Tringali A, Gkolfakis P (2025). Endoscopic retrograde cholangiopancreatography: a comprehensive review as a single diagnostic tool. Best Pract Res Clin Gastroenterol.

[5] Barakat M, Saumoy M, Forbes N, Elmunzer BJ (2025). Complications of endoscopic retrograde cholangiopancreatography. Gastroenterology.

[6] Guelrud M (2001). Endoscopic retrograde cholangiopancreatography. Gastrointest Endosc Clin N Am.

[7] Asenov Y, Akın M, Cantez S, Gün Soysal F, Tekant Y (2019). Endoscopic retrograde cholangiopancreatography in children: retrospective series with a long-term follow-up and literature review. Turk J Gastroenterol.

[8] Chen X, Zhang X, Gao R, Huang Y, Mao S, Wang B, Feng J (2024). Effect of alleviating preoperative anxiety on gastrointestinal function recovery after laparoscopic high ligation of the hernia sac in children with indirect inguinal hernia. Drug Des Devel Ther.

[9] Dai F, Zhang R, Deng R, Wang G, Guo H, Guo C (2023). Regular use of low-dose of opioids after gastrointestinal surgery may lead to postoperative gastrointestinal tract dysfunction in children: a Chinese national regional health center experience sharing. BMC Gastroenterol.

[10] Tan S, Wu G, Yu W, Li N (2016). [Research advance in causes of postoperative gastrointestinal dysfunction]. Zhonghua Wei Chang Wai Ke Za Zhi.

[11] Neu J (2007). Gastrointestinal development and meeting the nutritional needs of premature infants. Am J Clin Nutr.

[12] Harris J, Chorath K, Balar E, Xu K, Naik A, Moreira A, Rajasekaran K (2022). Clinical practice guidelines on pediatric gastroesophageal reflux disease: a systematic quality appraisal of international guidelines. Pediatr Gastroenterol Hepatol Nutr.

[13] Kong Q, Chen LM, Liu CY, Li W, Yin PH (2024). The effect of acupuncture on gastrointestinal recovery after abdominal surgery: a narrative review from clinical trials. Int J Surg.

[14] Su H, Wen Y, Kang D (2022). Application of refined nursing combined with comprehensive treatment of traditional Chinese and western medicine in gastrointestinal dysfunction after tumor operation. Evid Based Complement Alternat Med.

[15] Wang Y, Wang L, Ni X, Jiang M, Zhao L (2024). Effect of acupuncture therapy for postoperative gastrointestinal dysfunction in gastric and colorectal cancers: an umbrella review. Front Oncol.

[16] Ruan D, Li J, Liu J, Li D, Ji N, Wang C, Qu Y, Li Y (2021). Acupoint massage can effectively promote the recovery of gastrointestinal function after gynecologic laparoscopy. J Invest Surg.

[17] Zhang M, Chen J (2023). Effect of ear point burying combined with acupoint massage on recovery of gastrointestinal function after gastrointestinal surgery. Minerva Pediatr (Torino).

[18] Dehghan M, Malakoutikhah A, Ghaedi Heidari F, Zakeri MA (2020). The effect of abdominal massage on gastrointestinal functions: a systematic review. Complement Ther Med.

[19] Liu P, Qin Z, Wu S, Zeng W, Guo W (2024). Effect of external application of Rhubarb and Mirabilite combined with warm acupuncture on the recovery after laparoscopic appendectomy. Drug Eval 2024.

[20] Cai H, Du J, Luo C, Li S (2023). External application of mirabilite before surgery can reduce the inflammatory response and accelerate recovery in mild acute biliary pancreatitis. BMC Gastroenterol.

[21] Gao B, Ding Y, Wang X (2024). The effect of external application of mirabilite on the recovery of gastrointestinal function after laparoscopic appendectomy. Clin Med 2024.

[22] Chan AW, Boutron I, Hopewell S, Moher D, Schulz KF, Collins GS, Tunn R, Aggarwal R, Berkwits M, Berlin JA, Bhandari N, Butcher NJ, Campbell MK, Chidebe RC, Elbourne DR, Farmer AJ, Fergusson DA, Golub RM, Goodman SN, Hoffmann TC, Ioannidis JPA, Kahan BC, Knowles RL, Lamb SE, Lewis S, Loder E, Offringa M, Ravaud P, Richards DP, Rockhold FW, Schriger DL, Siegfried NL, Staniszewska S, Taylor RS, Thabane L, Torgerson DJ, Vohra S, White IR, Hróbjartsson A (2025). SPIRIT 2025 statement: updated guideline for protocols of randomized trials. Nat Med.

[23] Hu L, Liu Y, Li J, Wang L, Zeng Y (2019). Effect of external application of Glauber’s salt of umbilical cord and point massage of Zusanli on postoperative gastrointestinal function in patients undergoing laparoscopic hepatectomy. J Nurs (China).

[24] Hu S, Chen J, Zhou Q, Jiang L (2023). Influence of acupoint massage on gastrointestinal function recovery of patients after gynecologic laparoscopic surgery: a meta-analysis. Chin Evid-Based Nurs 2023.

[25] Huang L Standard acupuncture point localization by the World Health Organization. World Health Organization (WHO).

[26] Zhang Q, Zhu L, Ju Y (2025). Effect of enteroclysis with Dachengqi Decoction combined with external application of mirabilite in early enteral nutrition support of patients with moderate severe acute pancreatitis. Hebei J TCM 2025.

[27] Guo C, Zhang Y, Ding Q, Wang Q, Yang Y, Feng X (2021). Ethical guidance for pediatric clinical research. Chin J Clin Pharmacol 2021.

[28] Santángelo A, Scarpin A, Imaz F, Marino P, Vargas R, Cardona Bidart L, Darrigran S, Macías M, Sánchez de Loria J, Volonté P, Salgueiro F (2025). One-step endoscopic retrograde cholangiopancreatography and laparoscopic cholecystectomy: a safe strategy in pediatrics. Cir Pediatr.

[29] Qin XM, Yu FH, Lv CK, Liu ZM, Wu J (2023). Endoscopic retrograde cholangiopancreatography for diagnosing and treating pediatric biliary and pancreatic diseases. World J Gastrointest Surg.

[30] Sun R, Xu X, Zheng Q, Zhan J (2022). Therapeutic endoscopic retrograde cholangiopancreatography for pediatric hepato-pancreato-biliary diseases: a systematic review and meta-analysis. Front Pediatr.

[31] Kheir FA, Moustafa L, Ahmad L, Kamil H, Mahmod J, Sandouk F (2025). Duct-related complications of pediatric post-traumatic pancreatitis: a case report from Syria. Int J Surg Case Rep.

[32] Lorio E, Moreau C, Hernandez B, Rabbani T, Michaud K, Hachem J, Aggarwal P, Stolow E, Brown L, Michalek JE, Patel S (2023). Pediatric ERCP: factors for success and complication-a 17-year, multisite experience. J Pediatr Gastroenterol Nutr.

[33] Liu G, Zhang J, Zhao S, Li HJ (2025). Research progress on the treatment of gastrointestinal dysfunction after laparoscopic surgery using traditional Chinese medicine external treatment methods. China Mod Doctor.

[34] Zhang Y, Hou C, Tang S, Duan Y, Weng S (2020). The effect of acupuncture manipulation on gastrointestinal function recovery and gastrointestinal hormone levels after gynecological laparoscopic surgery. Mod J Integr Tradit Chin West Med.

[35] Wang C, Wang L, Xiang J, Sun W (2022). Protective effect and mechanism of mirabilite external application on gastrointestinal function in patients with sepsis complicated with gastrointestinal dysfunction. Chin Mod Doctor.

[36] Tao L, Fu J, Wang F, Song Y, Li Y, Zhang J, Wang Z (2023). The application of mirabilite in traditional Chinese medicine and its chemical constituents, processing methods, pharmacology, toxicology and clinical research. Front Pharmacol.

