# Supplementary material for: Combined Acupoint Massage and Abdominal Mirabilite Application for Accelerating Gastrointestinal Recovery in Pediatric Patients After Endoscopic Retrograde Cholangiopancreatography: Protocol for a Randomized Controlled Trial
Source: JMIR Res Protoc. 2026 Feb 3;15:e87961. doi: 10.2196/87961 (PMC12914232; doi:10.2196/87961)
Supplement: Multimedia Appendix 1 [file resprot_v15i1e87961_app1.docx]

**Post-operative application of mirabilite and acupoint massage after pediatric ERCP**

Version: 1.0

Applicable to: Trained and certified physicians and nurses

**Part One: Core Principles**

**1. Triple Safety Assurance**

**1.1 Pre-Operation Assessment**

Vital signs stable (HR 80-120 beats/min, SpO₂≥ 95%)

Abdominal wound without seepage/redness (operation at least 3 cm from the incision)

Confirm no history of hypersensitivity to mirabilite (skin test before first application: 0.1 ml of 10% mirabilite solution applied to the forearm for 15 minutes)

**1.2 Intra-Operation Monitoring**

Continuously ask the child about their feelings (use a facial pain scale for children ≤ 5 years old)

Check skin/tubing condition every 5 minutes

Equip with a first aid kit (including epinephrine and oxygen mask)

**1.3 Post-Operation Follow-Up**

Observe skin at the application site for at least 24 hours

No food for 30 minutes after massage to prevent vomiting

**2. Child Adaptation Guidelines**

**2.1 Ages 2-3**

Demonstrate the procedure using a doll and provide reward stickers

**2.2 Ages 4-7**

Use a story metaphor: “The little train in the tummy is about to start!”

**2.3 Ages 8-12**

Explain the scientific principles and allow self-timing

**Part Two: Standardized Procedure for the Application of Mirabilite**

1. **Environmental Preparation**

Room temperature: 25-27°C, play soothing music (≤50 decibels), 100% parental accompaniment rate.

1. **Material Preparation**

| Item | Specifications |
| --- | --- |
| Medical Mirabilite | Sodium sulfate purity ≥ 99%, sealed and stored dry |
| Warm Water | 35–38°C (feels slightly warm) |
| Sterile Gauze | 10cm × 10cm, 2 layers thick |
| Breathable Adhesive Tape | Low allergenic (e.g., paper tape) |
| Measuring Cup/Electronic Scale | Accurate to 0.1g |

1. **Operational Steps**

| Step | Key Points |
| --- | --- |
| 1 | Prepare paste: Mix Mirabilite powder with warm water in a 1:1 ratio (e.g., 50g Mirabilite + 50ml water) until smooth and free of granules. |
| 2 | Make the dressing: Evenly apply the paste onto gauze (thickness ≈ 0.5cm), covering the area around the navel (from above the xiphoid process to below the pubic symphysis). |
| 3 | Secure the dressing: Avoid drainage tubes, place gauze on the abdomen, and secure with adhesive tape in a “井” pattern (do not apply pressure to the abdomen). |
| 4 | Record Observations: - Start time: ________ - End time: ________ - Skin condition: Check for redness and rashes before, during, and after the application, and record. - Child’s tolerance: Quiet / Slight discomfort / Crying (note the reason). |
| 5 | Remove and clean: Remove gauze after 1 hour, clean skin with warm water, check and record skin condition. |

1. **Key Parameter Control**

| Variable | Standard Range | Handling Deviation |
| --- | --- | --- |
| Application Time | 60±5 minutes | If overdue → record reason and report to PI |
| Skin Temperature | 36-38°C, ΔT≤1°C | If elevated → pause and use cool compress |
| Adhesive Tape Pressure | ≤ 10mmHg (impression disappears in < 3s) | If too tight → replace with elastic mesh covering |

1. **Safety Warnings**

**5.1 Contraindications**

Skin lesions, history of allergic erythema, contamination of dressing area by drainage tube effusion.

**5.2 Abnormal Handling**

If skin redness/bullae occur → Immediately discontinue use → Apply calamine lotion → Report adverse event.

**Part Three: Standardized Procedures for Acupoint Massage**

1. **Anatomical Standards for Acupoint Localization**

| Acupoint | Surface Localization Method (Based on Bony Landmarks) | Verification Method |
| --- | --- | --- |
| Zusanli | Inferior edge of the tibial tuberosity → External 1 transverse finger + 3 transverse fingers below (child’s finger width) | Effective if the fibularis longus muscle contracts upon pressure |
| Tianshu | Intersection point of the umbilical plane and the clavicular midline | Pulsation can be felt at the outer edge of the rectus abdominis muscle |
| Zhongwan | Midpoint of the line connecting the xiphoid process and umbilicus | Deep pressure reveals pulsation of the abdominal aorta |
| Neiguan | Two transverse fingers from the proximal end of the wrist crease, in the groove between the palmaris longus tendon and the radial side wrist flexor tendon | Tendon becomes visible when the wrist is flexed |

1. **Massage Techniques and Procedure**

| Step | Key Operation Points |
| --- | --- |
| 1 | Position: Child lying flat, exposing abdominal and limb acupoints, ensuring warmth. |
| 2 | Techniques:  - Kneading: Use fingertips to draw circles clockwise (diameter 2 cm); intensity should result in slight redness of the skin, with no signs of pain from the child.  - Pressing: Apply downward pressure vertically (depth 0.5-1 cm), press for 1 second → relax → repeat. |
| 3 | Sequence: Zhongwan → Bilateral Tianshu → Bilateral Zusanli → Bilateral Neiguan. Each acupoint for 2 minutes (total duration ≈ 15 minutes). |
| 4 | Intensity Control:  - Ages 2-5: Fingertip pressure ≤ 0.5 kg (like lightly pressing an orange)  - Ages 6-12: Fingertip pressure ≤ 1 kg (like lightly pressing an apple) |
| 5 | Recording:  - Actual operation time: ________  - Child’s response: Cooperative / Slightly wriggling / Crying (specify acupoint). |

1. **Safety Warnings**
   Contraindications: Blood oozing from abdominal wounds, severe abdominal distension, continuous crying or resistance for more than 1 minute.
   Abnormal Handling: In case of sudden vomiting/abdominal pain during the procedure → Immediately stop → Assess vital signs → Report to the medical team.

**Part Four: Collaborative Intervention and Coordinated Management**

1. **Timing and Avoidance Rules**

Preferred Method: Massage → Interval ≥ 60 min → Application of mirabilite externally

Prohibited Method: Massage is prohibited within 1 hour after external application of mirabilite (to avoid skin irritation from salt crystallization)

1. **Recording Requirements**

Fill in the details of both procedures and the interval time in the "Collaborative Intervention Record Form."

| Acupoint Massage | Start Time：________ |
| --- | --- |
|  | End Time：________ |
|  | Duration：________ |
| External Application of Mirabilite | Start Time：________ |
|  | End Time：________ |
|  | Duration：________ |
| Interval Time | ________ |

**Part Five: Quality Assurance System**

1. **Qualifications of the Interveners**

Certified and trained doctors and nurses

1. **Operator Certification Standards**

Theoretical Assessment: Acupoint location error ≤ 3mm

Practical Assessment: Achieve effective intensity while enabling the child to accept it

Child Communication: Able to get 90% of target-age children to cooperate in completing the procedure

1. **Emergency Contact Network:**

| Event Level | Contact Person | Response Time |
| --- | --- | --- |
| Severe Allergy | Chief of Anesthesiology | 5min |
| Operation-related Injury | On-duty Surgeon | 10min |
