# Supplementary material for: Combined Acupoint Massage and Abdominal Mirabilite Application for Accelerating Gastrointestinal Recovery in Pediatric Patients After Endoscopic Retrograde Cholangiopancreatography: Protocol for a Randomized Controlled Trial
Source: JMIR Res Protoc. 2026 Feb 3;15:e87961. doi: 10.2196/87961 (PMC12914232; doi:10.2196/87961)
Supplement: Multimedia Appendix 2 [file resprot_v15i1e87961_app2.docx]

**SPIRIT 2025 Checklist for Trials**

Chan AW, Boutron I, Hopewell S, Moher D, Schulz KF, Collins GS, et al. SPIRIT 2025 statement: updated guideline for protocols of randomized trials. Nat Med. 2025 Jun;31(6):1784-92. PMID: 40295741. doi: 10.1038/s41591-025-03668-w.

| **Section/topic** | **No.** | **SPIRIT 2025 checklist item description** | **Page and Line Number** |
| --- | --- | --- | --- |
| Administrative information | | | |
| Title and structured summary | 1a | Title stating the trial design, population, and interventions, with identification as a protocol | Page 1, Line 1–3 |
|  | 1b | Structured summary of trial design and methods, including items from the World Health Organization Trial Registration Data Set | Page 1–2, Line 4–30 |
| Protocol version | 2 | Version date and identifier | Page 2, Line 36–37 |
| Roles and responsibilities | 3a | Names, affiliations, and roles of protocol contributors | Page 25, Line 523–527 |
|  | 3b | Name and contact information for the trial sponsor | ITMCTR2025000670 |
|  | 3c | Role of trial sponsor and funders in design, conduct, analysis, and reporting of trial; including any authority over these activities | Page 25, Line 517–518 |
|  | 3d | Composition, roles, and responsibilities of the coordinating site, steering committee, endpoint adjudication committee, data management team, and other individuals or groups overseeing the trial, if applicable | ITMCTR2025000670 |
| Open science | | | |
| Trial registration | 4 | Name of trial registry, identifying number (with URL), and date of registration. If not yet registered, name of intended registry | Page 2, Line 36–37 |
| Protocol and statistical analysis plan | 5 | Where the trial protocol and statistical analysis plan can be accessed | Page 7, Line 149–150 |
| Data sharing | 6 | Where and how the individual de-identified participant data (including data dictionary), statistical code, and any other materials will be accessible | Page 25, Line 520–521 |
| Funding and conflicts of interest | 7a | Sources of funding and other support (for example, supply of drugs) | Page 25, Line 517–518 |
|  | 7b | Financial and other conflicts of interest for principal investigators and steering committee members | Page 25, Line 529 |
| Dissemination policy | 8 | Plans to communicate trial results to participants, healthcare professionals, the public, and other relevant groups (for example, reporting in trial registry, plain language summary, publication) | ITMCTR2025000670 |
| Introduction | | | |
| Background and rationale | 9a | Scientific background and rationale, including summary of relevant studies (published and unpublished) examining benefits and harms for each intervention | Page 3–5, Line 41–103 |
|  | 9b | Explanation for choice of comparator | Page 5, Line 104–106 |
| Objectives | 10 | Specific objectives related to benefits and harms | Page 6–7, Line 121–137 |
| Methods: Patient and public involvement, trial design | | | |
| Patient and public involvement | 11 | Details of, or plans for, patient or public involvement in the design, conduct, and reporting of the trial | Page 6–20, Line 120–418 |
| Trial design | 12 | Description of trial design including type of trial (for example, parallel group, crossover), allocation ratio, and framework (for example, superiority, equivalence, non-inferiority, exploratory) | Page 6, Line 139–144 |
| Methods: Participants, interventions, and outcomes | | | |
| Trial setting | 13 | Settings (for example, community, hospital) and locations (for example, countries, sites) where the trial will be conducted | Page 6, Line 140–141 |
| Eligibility criteria | 14a | Eligibility criteria for participants | Page 8, Line 154–164 |
|  | 14b | If applicable, eligibility criteria for sites and for individuals who will deliver the interventions (for example, surgeons, physiotherapists) | Page 11, Line 224–226 |
| Intervention and comparator | 15a | Intervention and comparator with sufficient details to allow replication including how, when, and by whom they will be administered. If relevant, where additional materials describing the intervention and comparator (for example, intervention manual) can be accessed | Page 11–12, Line 223–256 |
|  | 15b | Criteria for discontinuing or modifying allocated intervention/comparator for a trial participant (for example, drug dose change in response to harms, participant request, or improving/worsening disease) | Page 16–17, Line 347–356 |
|  | 15c | Strategies to improve adherence to intervention/comparator protocols, if applicable, and any procedures for monitoring adherence (for example, drug tablet return, sessions attended) | Page 14, Line 289–293 |
|  | 15d | Concomitant care that is permitted or prohibited during the trial | Page 11, Line 219–222 |
| Outcomes | 16 | Primary and secondary outcomes, including the specific measurement variable (for example, systolic blood pressure), analysis metric (for example, change from baseline, final value, time to event), method of aggregation (for example, median, proportion), and time point for each outcome | Page 13–14, Line 268–287 |
| Harms | 17 | How harms are defined and will be assessed (for example, systematically, non-systematically) | Page 16, Line 331–341 |
| Participant timeline | 18 | Time schedule of enrollment, interventions (including any run-ins and washouts), assessments, and visits for participants. A schematic diagram is highly recommended (see Table 2) | Page 31, Table 1 |
| Sample size | 19 | How sample size was determined, including all assumptions supporting the sample size calculation | Page 9, Line 175–186 |
| Recruitment | 20 | Strategies for achieving adequate participant enrollment to reach target sample size | Page 8, Line 169–173 |
| Methods: Assignment of interventions | | | |
| Randomization: Sequence generation | 21a | Who will generate the random allocation sequence and the method used | Page 9, Line 188–190 |
|  | 21b | Type of randomization (simple or restricted) and details of any factors for stratification. To reduce predictability of a random sequence, other details of any planned restriction (for example, blocking) should be provided in a separate document that is unavailable to those who enroll participants or assign interventions | Page 9, Line 188–190 |
| Allocation concealment mechanism | 22 | Mechanism used to implement the random allocation sequence (for example, central computer/telephone; sequentially numbered, opaque, sealed containers), describing any steps to conceal the sequence until interventions are assigned | Page 9, Line 192–197 |
| Implementation | 23 | Whether the personnel who will enroll and those who will assign participants to the interventions will have access to the random allocation sequence | Page 9, Line 190–192 |
| Blinding | 24a | Who will be blinded after assignment to interventions (for example, participants, care providers, outcome assessors, data analysts) | Page 10, Line 199–210 |
|  | 24b | If blinded, how blinding will be achieved and description of the similarity of interventions | Page 10, Line 203–208 |
|  | 24c | If blinded, circumstances under which unblinding is permissible, and procedure for revealing a participant’s allocated intervention during the trial | Page 9-11, Line 187–216 |
| Methods: Data collection, management, and analysis | | | |
| Data collection methods | 25a | Plans for assessment and collection of trial data, including any related processes to promote data quality (for example, duplicate measurements, training of assessors) and a description of trial instruments (for example, questionnaires, laboratory tests) along with their reliability and validity, if known. Reference to where data collection forms can be accessed, if not in the protocol | Page 14–15, Line 295–306 |
|  | 25b | Plans to promote participant retention and complete follow-up, including list of any outcome data to be collected for participants who discontinue or deviate from intervention protocols | Page 14, Line 289–293 |
| Data management | 26 | Plans for data entry, coding, security, and storage, including any related processes to promote data quality (for example, double data entry; range checks for data values). Reference to where details of data management procedures can be accessed, if not in the protocol | Page 15–16, Line 307–322 |
| Statistical methods | 27a | Statistical methods used to compare groups for primary and secondary outcomes, including harms | Page 17–18, Line 358–386 |
|  | 27b | Definition of who will be included in each analysis (for example, all randomized participants), and in which group | Page 17, Line 360–361 |
|  | 27c | How missing data will be handled in the analysis | Page 17, Line 360–361 |
|  | 27d | Methods for any additional analyses (for example, subgroup and sensitivity analyses) | Page 18, Line 370–379 |
| Methods: Monitoring | | | |
| Data monitoring committee | 28a | Composition of data monitoring committee (DMC); summary of its role and reporting structure; statement of whether it is independent from the sponsor and funder; conflicts of interest and reference to where further details about its charter can be found, if not in the protocol. Alternatively, an explanation of why a DMC is not needed | ITMCTR2025000670 |
|  | 28b | Explanation of any interim analyses and stopping guidelines, including who will have access to these interim results and make the final decision to terminate the trial | Page 2, Line 28–29 |
| Trial monitoring | 29 | Frequency and procedures for monitoring trial conduct. If there is no monitoring, give explanation | Page 16–17, Line 324–341 |
| Ethics | | | |
| Research ethics approval | 30 | Plans for seeking research ethics committee/institutional review board approval | Page 19, Line 406–409 |
| Protocol amendments | 31 | Plans for communicating important protocol modifications to relevant parties | ITMCTR2025000670 |
| Consent or assent | 32a | Who will obtain informed consent or assent from potential trial participants or authorized proxies, and how | Page 8, Line 171–173 |
|  | 32b | Additional consent provisions for collection and use of participant data and biological specimens in ancillary studies, if applicable | ITMCTR2025000670 |
| Confidentiality | 33 | How personal information about potential and enrolled participants will be collected, shared, and maintained in order to protect confidentiality before, during, and after the trial | Page 15, Line 312–317 |
| Ancillary and post-trial care | 34 | Provisions, if any, for ancillary and post-trial care, and for compensation to those who suffer harm from trial participation | ITMCTR2025000670 |
