# Supplementary material for: Combined Acupoint Massage and Abdominal Mirabilite Application for Accelerating Gastrointestinal Recovery in Pediatric Patients After Endoscopic Retrograde Cholangiopancreatography: Protocol for a Randomized Controlled Trial
Source: JMIR Res Protoc. 2026 Feb 3;15:e87961. doi: 10.2196/87961 (PMC12914232; doi:10.2196/87961)
Supplement: Multimedia Appendix 3 [file resprot_v15i1e87961_app3.docx]

**Expert Opinions from the Proposal Meeting**

**Project Title**

Combined Acupoint Massage and Abdominal Mirabilite Application for Accelerating Gastrointestinal Recovery in Pediatric Patients after ERCP: A Randomized Controlled Trial Protocol

**Comprehensive Expert Evaluation Opinions**

This is a well-designed and highly promising study protocol addressing a clinically significant challenge in pediatric care. The investigation into acupoint massage and mirabilite application to accelerate gastrointestinal recovery post-ERCP in children is both innovative and timely. The study holds considerable potential to establish a safe, effective adjunctive therapy, reducing complications and hospital stays in this vulnerable population. However, the following aspects could be further improved:

**Q1: The method of randomization requires greater detail. Please specify the exact software used to generate the random allocation sequence.**

**Response:**

Thank you for your valuable suggestions regarding our project. We sincerely appreciate your suggestion to enhance the methodological rigor of our randomization process. As requested, we have revised "Randomization and blinding" to provide explicit details regarding the generation of the random allocation sequence. The revised text is as follows:

“This study employs a randomized block design with a block length of 4. Random numbers for grouping were generated using Stata 18.0 software. A dedicated researcher, who will not participate in the recruitment of participants or data collection, will oversee the random grouping process, thereby ensuring that the grouping decisions remain independent of actual operations. Prior to the initiation of the study, personnel not involved in participant recruitment or data collection will create an allocation table based on the random number sequence and maintain this table in strict confidentiality to prevent any potential leaks. Group allocations will be concealed within sealed opaque envelopes, which the researcher will open prior to the intervention in order to access the allocation plan for each patient. Although the characteristics of the treatment preclude the implementation of blinding, we will utilize evaluator blinding to mitigate the risk of implementation and measurement bias. Assessments will be performed by independent researcher who remains unaware of the participants’ allocations. ”

This revision has clearly specified the specific software version and has added details regarding randomization and allocation concealment. We believe this addition strengthens the reproducibility and validity of our trial design. Once again, we deeply appreciate your insightful comment, which has significantly improved the clarity of our methodology.

**Q2: The sample size calculation needs elaboration, please provide comprehensive details.**

**Response:**

Thank you for your thoughtful review and valuable feedback regarding the sample size calculation. We sincerely appreciate your expertise in highlighting this important methodological aspect, as it allows us to clarify our approach and strengthen the study's statistical rigor.

Our sample size calculation, performed using G*Power software, is based on detecting a clinically relevant medium effect size (f=0.40) in the primary outcome (time to GI recovery) across four groups using a one-way ANOVA, with α=0.05 and power=80%. The initial calculation yielded 76 participants. To account conservatively for a potential 20% dropout rate, the total target enrollment is 96 participants (24 per group). This sample size is deemed sufficient to address the study's primary aim of comparing the efficacy of the interventions while balancing statistical rigor with the practical realities of recruiting pediatric patients undergoing a specialized procedure like ERCP.

Thank you once again for your critical review, which has provided us the opportunity to clarify this essential methodological component. We believe this detailed explanation strengthens the protocol's transparency and statistical foundation.

**Q3: The descriptions of both the acupoint massage and external application of mirabilite interventions require significantly more detail. Provide a complete, step-by-step protocol covering all aspects of application (e.g., techniques, pressure, duration, frequency, specific locations on the abdomen for mirabilite, preparation method/form of mirabilite used). Ideally, include the full interventionist manual/protocol as supplementary material to ensure clarity, consistency, and reproducibility.**

**Response:**

Thank you for your valuable feedback and insightful request for greater methodological detail regarding the acupoint massage and mirabilite application interventions. We sincerely appreciate the opportunity to enhance the clarity, reproducibility, and rigor of our protocol. We have developed a detailed Interventionist Manual (Supplementary Material 2), ensuring strict standardization across all operators. We believe the dedicated supplementary manual, fully address your request for enhanced detail, ensuring the interventions are precisely described, consistently applied, and readily reproducible. Thank you again for your meticulous review, which has significantly strengthened the methodological rigor of our study protocol.

**Q4: The collection of general participant information should be expanded.**

**Response:**

Thank you very much for your valuable suggestion regarding the collection of general participant information. We sincerely appreciate your insightful comment, which will undoubtedly help improve the quality and comprehensiveness of our study. According to your suggestion, we expand the scope of collecting participants' basic information. The additional content of the data is as follows:

“To characterize the study population and assess potential confounding factors, we will collect basic demographic and clinical information from pediatric patients prior to the intervention, including: age, gender, height, weight, and body mass index, as well as disease diagnosis, specific indications for undergoing ERCP, history of previous abdominal surgeries, comorbidities, relevant family medical history, the number of prior ERCP procedures, guardians’ educational level, annual family income (categorized as <50,000, 50,000-100,000, 100,000-200,000, and >200,000 RMB), and type of residence (urban or rural). ”

We believe these additional data points will provide valuable context for interpreting our primary outcomes and allow for more thorough subgroup analyses. The expanded dataset will help us better characterize our study population and identify potential confounding factors that might influence gastrointestinal recovery.

**Q5: The justification for the selection of specific acupoints used in the Tuina intervention needs to be explicitly stated. Provide the theoretical basis (e.g., TCM principles, meridian theory, existing literature) or empirical reasoning for choosing these particular points.**

**Response:**

Thank you for your insightful question regarding the acupoint selection for our Tuina intervention. We deeply appreciate your expertise and the opportunity to clarify this critical aspect of our study design.

The selection of acupoints—Zusanli (ST-36), Tianshu (ST-25), Zhongwan (CV-12), and Neiguan (PC-6)—is grounded in both Traditional Chinese Medicine (TCM) theory and empirical evidence from modern research. In TCM, postoperative gastrointestinal dysfunction is attributed to "Qi stagnation" and "bowel obstruction," and these acupoints were chosen to regulate Qi, unblock meridians, and restore visceral function. Specifically:

Zusanli (ST-36),the "He-Sea" point of the Stomach Meridian, renowned for strengthening spleen-stomach function and promoting peristalsis.

Tianshu (ST-25), the Front-Mu point of the Large Intestine, directly addresses abdominal distension and constipation by regulating intestinal Qi.

Zhongwan (CV-12), the Front-Mu point of the Stomach and a key intersection of the Conception Vessel, harmonizes middle-jiao Qi to alleviate nausea and bloating.

Neiguan (PC-6), a Luo-connecting point, calms the stomach and stops vomiting by regulating the Pericardium Meridian’s connection to the Sanjiao.

These points are further supported by clinical studies. For example, Ruan et al. (2021) demonstrated that ST-36 and PC-6 significantly accelerated gastrointestinal recovery post-laparoscopy by enhancing vagal activity and motilin secretion. Similarly, Zhang et al. (2023) validated CV-12 and ST-25 for reducing postoperative ileus in pediatric abdominal surgery. Our protocol aligns with the WHO Standard Acupuncture Point Localization to ensure reproducibility.

We sincerely appreciate your guidance, which has allowed us to elaborate on this foundational aspect of our intervention. Your feedback underscores the importance of bridging TCM theory with contemporary evidence, and we hope this clarification strengthens the methodological rigor of our study.

References Cited in Response:

1. Ruan D, Li J, Liu J, Li D, Ji N, Wang C, Qu Y, Li Y: Acupoint Massage Can Effectively Promote the Recovery of Gastrointestinal Function after Gynecologic Laparoscopy. J Invest Surg 2021, 34(1):91-95.
2. Zhang M, Chen J: Effect of ear point burying combined with acupoint massage on recovery of gastrointestinal function after gastrointestinal surgery. Minerva Pediatr (Torino) 2023, 75(5):768-770.
3. Huang L: Standard acupuncture point localization by the World Health Organization: People's Health Publishing House.

**Q6: A formal plan for managing any potential adverse events or reactions related to the interventions is absent. Develop and include a clear emergency response protocol detailing the steps to be taken if AEs/ARs occur (e.g., types of reactions anticipated, assessment procedures, specific management actions, criteria for discontinuation, reporting procedures).**

**Response:**

Thank you for your insightful feedback regarding the safety monitoring and adverse event management plan. We sincerely appreciate your emphasis on participant safety, which is a cornerstone of our study. Below, we outline the formal emergency response protocol integrated into our revised manuscript (“Quality control and precautions”), with further clarifications:

“Emergency response plans have been pre-established to address potential safety issues. To manage possible skin allergic reactions resulting from the external application of mirabilite, emergency medications will be readily available on-site for localized treatment; should a clear allergic reaction or severe skin irritation arise, the application of mirabilite will be immediately terminated, symptomatic treatment will be administered, and adverse events will be reported as required. If a child exhibits significant discomfort or resistance during acupoint massage, the operator should promptly reduce the intensity or suspend the procedure, and if necessary, completely terminate the acupressure intervention for that session, documenting the reasons. All research personnel must be thoroughly familiar with and strictly adhere to these safety protocols and emergency plans, prioritizing the safety of the children involved and promptly reporting any incidents that exceed the prescribed protocols or severe adverse events to the principal investigator and ethics committee.”

We deeply value your expertise, which has strengthened our protocol’s rigor. Your critique ensures our study aligns with international safety standards, ultimately safeguarding pediatric participants. Should further refinements be needed, we welcome your guidance.
